# Advances in Engineered Nano-Biosensors for Bacteria Diagnosis and Multidrug Resistance Inhibition

**DOI:** 10.3390/bios14020059

**Published:** 2024-01-23

**Authors:** Qingxiu Xia, Hui Jiang, Xiaohui Liu, Lihong Yin, Xuemei Wang

**Affiliations:** 1 Key Laboratory of Environmental Medicine Engineering, Ministry of Education, School of Public Health, Southeast University, Nanjing 210009, China; 220213995@seu.edu.cn; 2 State Key Laboratory of Digital Medical Engineering, School of Biological Science and Medical Engineering, Southeast University, Nanjing 210096, China101013182@seu.edu.cn (X.L.)

**Keywords:** bacteria, multidrug resistance (MDR), nano-biosensors, bacteria theranostics

## Abstract

Bacterial infections continue to pose a significant global health challenge, with the emergence of multidrug-resistant (MDR) bacteria and biofilms further complicating treatment options. The rise of pan-resistant bacteria, coupled with the slow development of new antibiotics, highlights the urgent need for new therapeutic strategies. Nanotechnology-based biosensors offer fast, specific, sensitive, and selective methods for detecting and treating bacteria; hence, it is a promising approach for the diagnosis and treatment of MDR bacteria. Through mechanisms, such as destructive bacterial cell membranes, suppression of efflux pumps, and generation of reactive oxygen species, nanotechnology effectively combats bacterial resistance and biofilms. Nano-biosensors and related technology have demonstrated their importance in bacteria diagnosis and treatment, providing innovative ideas for MDR inhibition. This review focuses on multiple nanotechnology approaches in targeting MDR bacteria and eliminating antimicrobial biofilms, highlighting nano-biosensors via photodynamics-based biosensors, eletrochemistry biosensors, acoustic-dynamics sensors, and so on. Furthermore, the major challenges, opportunities of multi-physical-field biometrics-based biosensors, and relevant nanotechnology in MDR bacterial theranostics are also discussed. Overall, this review provides insights and scientific references to harness the comprehensive and diverse capabilities of nano-biosensors for precise bacteria theranostics and MDR inhibition.

## 1. Introduction

### 1.1. Traditional Techniques

In recent years, statistics have shown that a large proportion of human illness and death is caused by bacterial diseases [1] and that the damage caused by bacterial biofilms and bacterial resistance is a major burden on the social economy and public health [2]. Antimicrobial resistance (AMR) poses a major threat to human health around the world. In 2019, AMR infections caused 1.27 million deaths directly and 4.95 million deaths indirectly, estimated for 204 countries and territories worldwide. By 2050, about 10 million additional direct deaths are expected each year, which is equivalent to the number of people who died from cancer globally in 2020 [3,4,5,6]. As a survival strategy, most bacteria will form a biofilm under suitable conditions. Bacteria in the biofilm state are more resistant to drugs than those in the free state, which is one of the major causes of clinical refractory infections. Although bacterial resistance has been widely reported, its association with bacterial biofilms is rarely discussed. At the same time, traditional techniques for bacterial diagnosis and therapy are outdated [7,8,9], such as smear microscopy, isolation culture and biochemical reaction and, histiocytoculture. They are cumbersome, time-consuming, and have low sensitivity and specificity, resulting in frequent clinical misdiagnosis.

### 1.2. Nano-Biosensor Techniques

**Figure 1:**
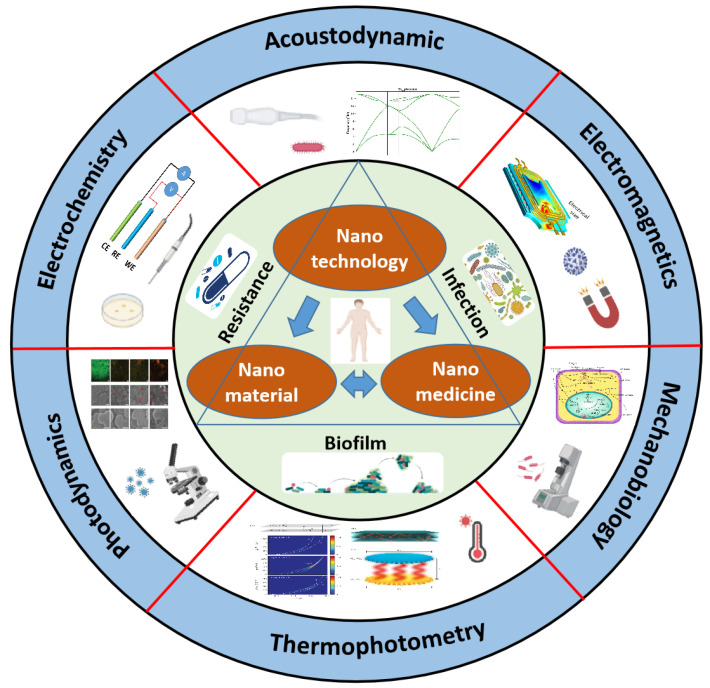
An overview of the multiple nanotechnology approaches for the diagnosis and treatment of bacteria.

With the rapid development of molecular biology technology, nanotechnology has emerged and tends to mature in continuous iterations. It has been extensively applied in the scientific research community, especially in the fields of environmental health monitoring, food safety management, and biomedical engineering, and has outstanding comprehensive advantages in combating bacterial infections, bacterial resistance, and bacterial biofilms [10]. Nanotechnology has unique physical, chemical, and biological characteristics, and it stands out in many diagnostic and therapeutic methods [11,12,13]. Due to the wide application of nanomaterials and nanotechnology in bacteria diagnosis and treatment, this article will focus on the recent research progress of engineered nano-biosensors for bacteria theranostics based on photodynamics, electrochemistry, acoustic dynamics, electromagnetism, photothermal, mechanobiology, and so on, as illustrated in Figure 1.

## 2. Bacterial-Related Infection

### 2.1. Bacterial Infection

At present, it is generally believed that bacteria will continue to grow, reproduce, release toxic substances, and other processes once they enter the body. It can cause various degrees of pathological changes in the body called bacterial infection or sometimes affects bacterial pathogeneses due to bacterial intoxication. Bacterial infections often manifest in two modes: acute and chronic. Different infectious bacteria often have different effects on the host body [14]. One is that in acute infection, bacteria cause acute inflammation in the host, usually shown as redness, swelling, warmth, pain, and functional limitations. The other is that in chronic infection, bacteria tend to gradually form biofilms over time and have a greater tolerance to the antimicrobial agents as well as to the host immune system. Self-limiting bacterial infections also exist. However, most bacterial infections are not very easy to treat [15]. The final formation of bacterial infection can be determined by both human immunity and bacterial pathogenicity. The outcome of bacterial infection depends on many factors that can influence the course, such as infection dosage, environmental factors, co-infections, etc.

### 2.2. Bacterial Resistance

It is well known that antibiotics select resistant bacteria, and, afterwards, the selected resistant strains become predominant. Bacteria that are resistant to antibiotics are becoming more and more common, creating a global health emergency that is difficult or impossible to treat. First, it is important to understand that antibiotic resistance is a natural and inevitable phenomenon. For billions of years, bacteria have struggled to evolve to constantly resist the effects of antimicrobial agents. Similarly, both external and internal causes contribute to the development of bacterial resistance. External causes refer to environmental mechanisms, i.e., the driving role of long-term ecological evolution in the rapid spread of bacterial drug resistance. The role of the environment can provide a means for bacterial colonization or host infection, causing changes in the DNA sequence occasionally, including genetic transfer between bacterial species [16,17].

### 2.3. Bacterial Biofilm

**Figure 2:**
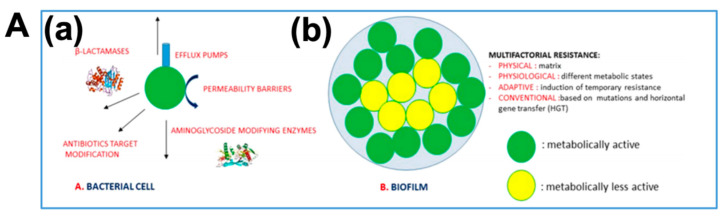

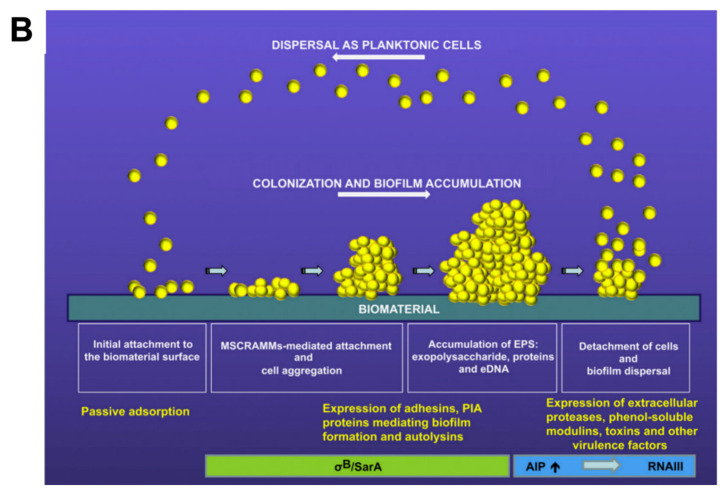
Resistance mechanisms at cellular level (**a**) and at community level (**b**) [17] (**A**). The cycle of biofilm [20] (**B**).

In the meantime, the formation of bacterial biofilms is a complex, dynamic, and continuous stratification process that could be divided into four steps: colonization, aggregation, maturation, and dissemination. (1) Colonization step: Cell surface charge, van der Waals force, hydrophobicity, and electrostatic force could help microorganisms to reversibly adhere to the covering of objects [18]. (2) Aggregation step: microorganisms swim in the liquid or aggregate on the solid surface to form microcolonies by synthesizing rotating flagella [19]. (3) Maturation step: mature biofilm microcolonies are surrounded by water transport channels, which can transport nutrients, enzymes, metabolites, and waste products. A large number of microorganisms grow, settle, and label on the appearance of living or inanimate substances to generate quorum sensing and gene regulation [20]. (4) Dissemination step: bacteria alternate between a floating state and a biofilm state attached to a multicellular community. The mature biofilm helps the microorganisms under the membrane to transfer energy, substances, and messages; at the same time, it can resist the harsh environment of the microorganisms under the biofilm [21]. In fact, the formation of bacterial biofilms implies a state of protected growth that not only allows cells to survive in harsh environments but also allows them to disperse into new niches [22,23]. Bacterial concentration, existence time, temperature, fluid dynamics, nutrient concentration, and physical and chemical properties of surface materials have positive or negative effects. Additional structures, such as flagella and fimbriae, on the bacterial surface also affect the formation of biofilms (Figure 2).

## 3. Engineered Nano-Biosensors for Precise Bacterial Diagnosis and Treatment

### 3.1. Photodynamics-Based Nano-Biosensors

Photodynamic therapy (PDT) has emerged as a new precision bacterial therapy method, with the advancement of optical technology, and the development of new photosensitizers has attracted wide attention in recent years. This is a new antibacterial agent, which induces them to produce reactive oxygen species (ROS) to kill bacteria. Nanotechnologies for bacterial diagnosis and therapy based on photodynamics are popular due to their low side effects and low drug resistance [24,25,26]. PDT is a promising therapeutic strategy for the treatment of bacterial infections.

#### 3.1.1. Phototherapeutic Drug Nanomaterials

Phototherapeutic drug materials have great therapeutic potential. Nanotechnology can be used to create multifunctional structures with targeted cytotoxicity and the ability to select markers. Combined, the two open up completely new possibilities, for example, against multidrug-resistant bacteria [27]. Researchers use raw materials from zeolith L-nanocrystals. The nanometer-sized particles attach a chemical compound to the bacterial coat in a very simple and inexpensive way. These particles can also contain dye molecules that glow green under a fluorescence microscope, making the bacteria clearly visible. Photodynamic processing refers to a reaction that occurs when light hits bacteria and kills them. Some researchers have attached a third substance to micron-sized crystals, which could be activated using red light and then produce certain reactive oxygen species molecules. These oxygen molecules, also including single oxygen, start the cascade effect that damages the bacterial cell [28].

#### 3.1.2. Photodynamic Combined Treatment

Researchers are developing a hypoxia-potentiating strategy by combining PDT and the prodrug metronidazole (MNZ) to treat bacterial biofilm infections. Hyaluronic acid (HA) was functionalized with chlorin e6 (Ce6) and MNZ to form HA-Ce6-MNZ nanoparticles (HCM NPs). After delivery to sites infected with *methicillin-resistant Staphylococcus aureus* (MRSA) biofilm, HCM NPs are degraded by MRSA-secreted hyaluronidase (Hyal) to release Ce6 and MNZ. On the basis of photodynamic therapy and atmospheric pressure conditions, laser irradiation of Ce6 could produce a reaction that generates 1O_2_, thus killing bacteria in the biofilm. Due to the consumption of O_2_ from PDT, the hypoxia of the biofilm is enhanced soon afterwards, promoting the production of nitro reductase from MRSA, which, furthermore, reduces the activation of MNZ and kills bacteria in the conditions of hypoxia [29]. This combination of photodynamic therapy can not only improve the hypoxic microenvironment but also eradicate the MRSA biofilm under normal oxygen conditions, induce the anaerobic metabolism of MRSA, and activate the antimicrobial activity of metronidazole.

#### 3.1.3. Nano-Biosensors upon NIR Light Irradiation

Nowadays, many organic or inorganic near-infrared nanomaterials have been used in the treatment of bacterial resistance. Some researchers have creatively developed nitric oxide (NO)-assisted PDT nanocomposite films for near-infrared irradiation. Hierarchically structured nanoparticles (UCNP@PCN), composed of upconversion nanoparticles (UCNPs) and porphyrinic MOFs (PCN-224), are initially prepared. After doping with L-arginine (LA), the particles were attached to a polyvinylidene fluoride (PVDF) matrix, and the electrospun nanocomposite membrane (UCNP@PCN@LA-PVDF) was obtained. ROS production is promoted under near-infrared light irradiation (980 nm). In addition to playing a bactericidal role in photodynamic therapy, ROS can induce loaded LA to produce NO, thus completing creative antibacterial behavior of NO-assisted PDT [30]. At the same time, this method of combining near-infrared light to irradiate nanoparticles can realize its potential in practical applications in biomedical engineering biosensors for bacteria recognition and treatment.

#### 3.1.4. Nanocage-Based Biosensor for Targeted Phototherapy

**Figure 3:**
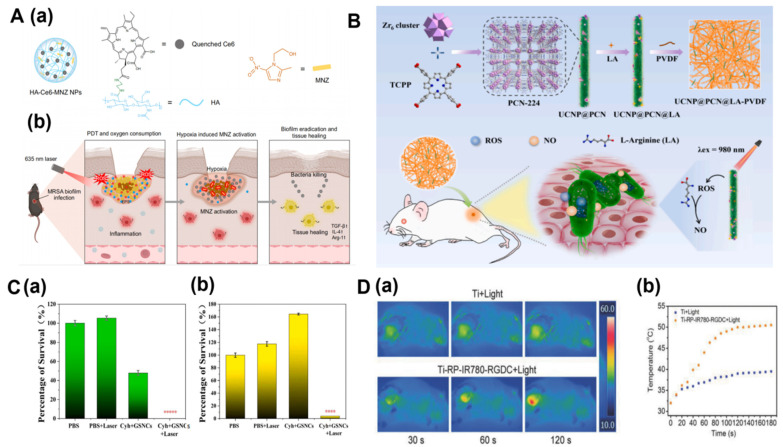
Potentiating hypoxia by PDT for antibiotic activation to combat MRSA biofilm infections [29] (**A**). Schematic illustration of the preparation and bactericidal activity of UCNP@PCN@LA-PVDF nanocomposite membrane [30] (**B**). The survival rate of antibacterial combination of GSNCs under different conditions for (**a**) MDR. *E. coli* and (**b**) MRSA. The bacterial survival data were obtained by plate counting method (Legend: **** *p* < 0.001; ***** *p* < 0.0001) [31] (**C**). Thermal images (**a**) and (**b**) temperature changes of Ti + Light and Ti-RP-IR780-RGDC + Light under 808 nm laser irradiation (2.0 W cm^−2^) [32] (**D**).

In addition, targeted phototherapy technology activates microenvironmental regulation of the antimicrobial activity of drugs through photodynamic therapy, and targeted phototherapy is often combined with nanomaterials to treat bacterial biofilms. In the past, metal nanomaterials, such as gold and silver, have been widely studied because of their unique local surface plasmon resonance (LSPR) properties. Due to their excellent stability, biosafety, and flexible modifiability, gold and silver nanomaterials have been widely used in high-sensitivity detection, imaging, and bacterial diagnosis and treatment. Qin developed an alternative method based on the aggregation-induced photothermal (AIP) effect, combining high silver-loaded gold silver nanocages (GSNCs) with thiolate. The synergistic therapy of GSNCs’ rapid silver release and near-infrared thermal effect could effectively remove the biofilm secreted by MDR bacteria in vitro and eliminate MDR *Staphylococcus aureus* (*S. aureus*) in disease-damaged mice. It can be seen that this method may be a new path to fight against refractory MDR bacterial infections [31]. Tan proposed to use red phosphorus and near-infrared to rapidly eradicate biofilms on phototube implants because red phosphorus has good biocompatibility and highly efficient photothermal ability [32] (Figure 3).

### 3.2. Electrochemistry-Based Nano-Biosensors

The use of bioelectric nanotechnology methods to kill and eradicate bacterial resistance and bacterial biofilms has attracted wide attention, particularly the identification of surface modification strategies that disrupt the bioelectric balance of intracellular and extracellular components of bacteria [33,34,35]. In addition, the inherent superiorities of electrochemical sensors, such as low cost, low power consumption, no complicated robotization, and microminiaturization, mean that they have the advantages of the rapid and quantitative detection of bacteria, particularly through the integration of microfluidic technology and nanotechnology for the on-site, rapid, and highly sensitive detection of complex bacterial samples. In addition, electrochemical detection is easily miniaturized and portable, which makes it very suitable for rapid and low-cost detection of bacterial disturbances in some areas with poor medical resources to avoid major public health events caused by detection delays.

#### 3.2.1. Custom-Designed Electrochemical Cell

Researchers have compared several pathogens, looking primarily at the effects of different types of antibiotics. Electrochemical cells could be constructed to detect MDR in bacteria. The starting point is a custom electrochemical cell with a glass carbon electrode. The bacterial culture is then added to the custom battery. At the same time, the growth medium is added to the soluble electron transfer medium, phenazine methosulfate (PMS). With cell respiration, the electrons released reduce the PMS, and the PMS, thus, oxidizes on the electrode surface, recording the current as it occurs. Fortunately, the results of the electrochemical antibiotic sensitivity test showed that strains were consistently categorized as either antibiotic resistant or sensitive, not only in <90 min of methodological development but also in <150 min of blind testing. It is believed that the detection time will be further shortened in the future [36]. This work demonstrates the development and validation of antibiotic susceptibility tests using electrochemical diagnostic techniques for the rapid classification of antibiotic-sensitive and drug-resistant pathogens.

#### 3.2.2. Three-Dimensional Electrode Scaffold

Three-dimensional electrode scaffolds’ mechanism of action is used for electrochemical coupling with intracellular metabolism and extracellular redox transformation. Bacteria in the three-dimensional electrode holder can generate a reference current density. In solar-powered biochemical pathways, three-dimensional electrode scaffolds can be added. The three-dimensional electrode scaffold platform is all-purpose and can be used in sustainable chemical production while closely linking the intrinsic physiological functions of bacteria. Similarly, it can also be called a semi-biological system. Because of its presence, the porous hydrophilic IO-ITO electrode structure is no longer alone and is integrated by electroactive bacteria [37]. This could also enable the real-time monitoring of bacterial infections.

#### 3.2.3. Nanopore Electrical Evaluation

Researchers have proposed a novel methodology for electrical monitoring using nanoporous alumina membranes of virulence factors given off by bacterial pathogens. Bacterial hyaluronidase (HYAL), generated by overly aggressive Gram-positive bacteria, was chosen for the modelling complex to test this idea. This electrochemical setup makes effective use for flat covering in indium tin oxide/poly (ethylene terephthalate) (ITO/PET) electrodes for their assembly with a nanoporous membrane. This method was monitored based on current changes caused by the formation of antibody–HYal immune complexes that block nanochannels, with a detection limit as low as 64 UI/mL (17.3 U/mg) HYAL [38]. This opens the way for the next application of the developed monitoring system to evaluate the anti-toxic potential of various complexes. The label-free approach is fast and inexpensive and avoids the use of time-consuming sandwich tests to monitor bacterial virulence/invasion and to test new antibacterials/antiagents.

#### 3.2.4. Microfluidic Impedance Biosensors

**Figure 4:**
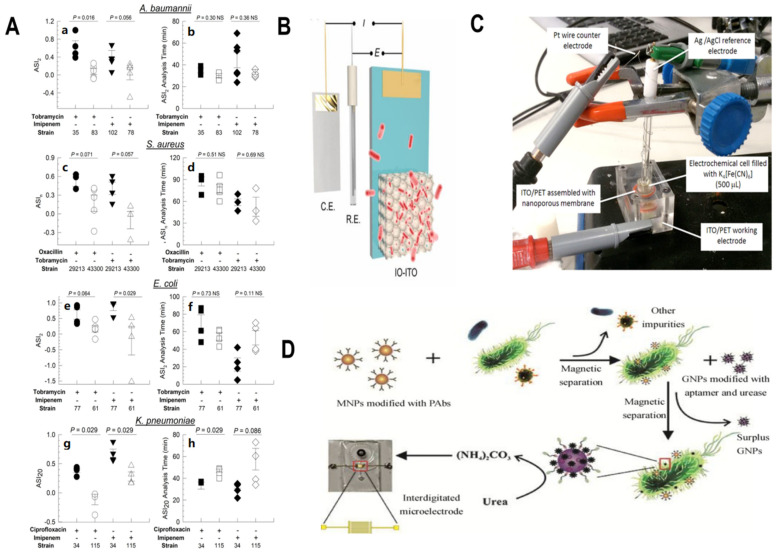
Antibiotic susceptibility index and analysis time. ASI_2_ (**a**) and ASI_2_ Analysis Time (**b**) for *A. baumannii* treated with or without 32 μg/mL tobramycin or 16 μg/mL imipenem (*n* > 4). ASI_10_ (tobramycin) or ASI_20_ (oxacillin) (**c**) and ASI_10_ (tobramycin) or ASI_20_ (oxacillin) Analysis Time (**d**) for *S. aureus* treated with 160 μg/mL tobramycin (*n* > 3) or 120 μg/mL oxacillin (*n* > 3). ASI_2_ (**e**) and ASI_2_ Analysis Time (**f**) for *E. coli* treated with 32 μg/mL tobramycin (*n* > 4) or 8 μg/mL imipenem (*n* = 4). ASI_20_ (**g**) and ASI_20_ Analysis Time (**h**) for *K. pneumoniae* treated with 20 μg/mL ciprofloxacin or 80 μg/mL imipenem (*n* = 4). Legend: black: Susceptible; white: Resistant species; NS not significant. Error bars represent standard error [36] (**A**). Inverse opal-indium tin oxide (IO-ITO) electrode as a platform for microbial electrogenesis and electrosynthesis using *G. sulfurreducens* [37] (**B**). Pictures of the experimental setup for the bacteria culture and electrochemical detection of secreted HYAL [38] (**C**). The principle of the microfluidic impedance biosensor based on immunomagnetic separation and urease catalysis for continuous-flow detection of *E. coli* O157:H7 [39] (**D**).

Combining immunomagnetic nanoparticles (MNPs) for bacterial isolation, urease for biosignal amplification, and microfluidic chips for electrochemical impedance sensing, researchers developed a microfluidic impedance biosensor for rapid, sensitive, and sustained flow monitoring in *E. coli* O157:H7. The relative impedance change rate of the sensor was linearly correlated with *E. coli* O157:H7 densities in 101 and 105 CFU/mL. *E. coli* O157:H7 concentrations as low as 1.2 × 101 CFU/mL could be detected within 2 h [39]. Interestingly, Yang introduced the ATP-binding box (ABC) transporter pathway to allow various bacteria to eat the gold nanoparticle autonomously, provided that the gold nanoparticle had been modified with a glucose polymer (GP). It is then irradiated by a laser, thereby mediating aggregation in the bacterial cells. The method was clear, and about 107 CFU bacteria residing in tumors or the gut could indeed be detected. This combination between imaging detection and comprehensive treatment is very important. This technique enables the visualization and therapy of diverse bacteria, which is a crucial step forward in the study of microbial ecosystems [40] (Figure 4).

### 3.3. Acoustic-Dynamics-Based Nano-Biosensors

In fact, sonodynamic therapy has its origins in photodynamic therapy, which primarily uses low-frequency ultrasound to excite sensitizers to produce ROS. Sonodynamic therapy (SDT) uses the ultrasonic (US) activation of acoustic sensitizers to produce ROS, which is highly cytotoxic to a variety of multidrug-resistant bacteria and does not develop resistance [41,42]. As a non-invasive therapy mode, ultrasound has good prospects for clinical application due to its inherent high tissue penetration power, which can break through the deep barriers [43,44]. At present, the mechanism of sonodynamic therapy is not conclusive, but the mainstream view shows that ROS is currently recognized as the main effector of sonodynamic therapy, regardless of the mechanism. As a special and novel technology, antimicrobial SDT (aSDT) displays unique potential in combating bacterial infections.

#### 3.3.1. Ultrasound-Switchable Nanozyme System

As the name implies, the nanoplatform (Pd@Pt-T790) is formed from an enzyme-catalyzed Pd@Pt nanoplate closely linked to tetra-(4-carboxyphenyl) porphyrin (T790) in an organic acoustic sensitizer. Due to the unique nature of its conversion, it can be controlled during ultrasonic activation, and it could effectively generate not only catalytic oxygen but also acoustic sensitizer-mediated reactive oxygen species, which are continuously accumulated, thereby reducing the anoxic-related barrier and, indeed, improving the efficacy of SDT. The advantages of this new ultrasound-switchable nanozyme system are numerous, including non-passive, non-fixed, and non-fuzzy characteristics, with significantly enhanced acoustic dynamics to eradicate deep-rooted bacterial infections [45]. Utilizing this US-switchable enzyme activity, significant accumulation at the site of infection, and excellent biocompatibility, Pd@Pt-T790-based SDT nanosystems have been successfully applied to eliminate MRSA-induced myositis and non-invasively monitor the progress of sonodynamic therapy via both photoacoustic imaging and magnetic resonance imaging. The developed US-switchable nanoenzyme system represents a promising strategy for active, controlled, and precise sonodynamic enhancement to eradicate deep bacterial infections.

#### 3.3.2. Low-Frequency Ultrasonic Sterilization

Compared with photodynamic technology, sonodynamic technology uses ultrasound as a means of stimulation, which has a good penetration depth to body tissues and can offset the shortcomings brought by photodynamic therapy. Different from photodynamic technology, the excitation source used in acoustic power is low-frequency ultrasound of 20 kHZ–3 MHz, and the penetration depth of soft tissue is up to 10 cm, which can effectively act on deep lesions in the body. Low-frequency ultrasound refers to ultrasound in a frequency range of 20 kHz–1 MHz [46]. With the continuous in-depth exploration of ultrasound, studies have shown that low-frequency ultrasound has strong penetration, high targeting ability, and remarkable effects, and it can achieve the destruction of bacterial biofilms and the killing of bacteria. Liu discovered, despite use in LFU (40 kHz, 600 mW/cm^2^, 30 min, duty cycle 1:9), individual or united with the single agent, a notable reduction in bacteria counts in biofilms, markedly promoting their anti-microbial effect. What’s more, higher densities of colistin in union therapies led to a superior ultrasound-enhanced antimicrobial effect. In 24 h time—kill curves, the combination of colistin (8 mg/mL) plus vancomycin (4 mg/mL) with LFU generated an obvious decrease in the total number of bacteria within biofilms after 8 h and a unremitting drop off till 24 h [47].

#### 3.3.3. Ultrasound-Activated Chemokinetic Therapy

Ultrasound-activated chemokinetic therapy (SCDT) is a new ultrasound-driven therapy approach. Due to the excellent characteristics of SCDT, it can not only perform non-invasive operation but also penetrate deep tissues. This is a highly effective way to combat bacterial resistance as well as bacterial infection. Superoxide anions with damaging hydroxyl radicals are produced through a catalytic reaction triggered by ultrasound [48]. The SCDT open platform was set out through adding Fe^3+^ onto polyethylenimide-modified Bismuth oxybromide (BiOBr) nanoplates. At the same time, the holes (h+) as well as electrons (e−) of BiOBr NPs were efficiently isolated during ultrasonic catalysis. Because the electron transport pathway is cut off and disrupted, the redox and Fenton reactions start, and too many excessive reactive oxygen species are produced, which can effectively compete with MRSA infection [49].

#### 3.3.4. Antibacterial Sonodynamic Nanocapturer

**Figure 5:**
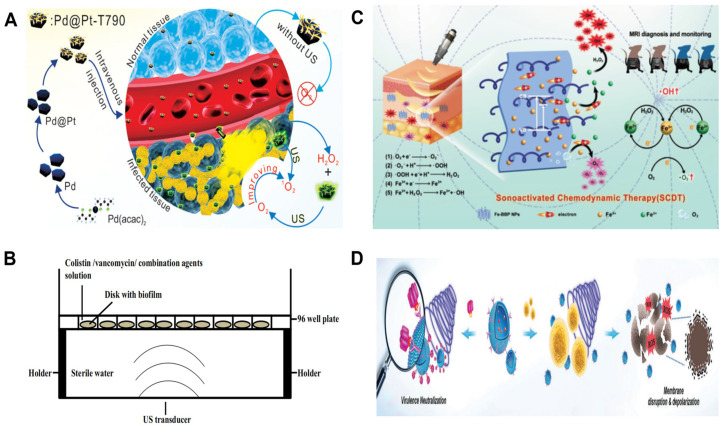
Diagrammatic drawing of the action mechanism of Pd@Pt-T790-mediated SDT [45] (**A**). Diagram depicting use of low-frequency ultrasound on biofilm treated with antibiotics [47] (**B**). Abridged general view on sonoactivated chemodynamic therapy against deep MRSA infection [48] (**C**). Schematic illustration of the antivirulence and antibacterial mechanism of ANVs [50] (**D**).

Pang proposed a simple and bioinspiring strategy to bridge antibacterial acoustic-dynamic therapy and antivirulent immunotherapy. As a proof of concept, an antibody neutralizing the alpha-toxin of MRSA was engineered onto the surface of the cell membrane nanovesicles, which was then encapsulated with a sound-sensitive agent. Compared to conventional passive virulence absorption using natural red blood cell (RBC) membranes, the highly active antiboil–toxin interaction enables the nanovesicles to capture virulence more effectively in vitro. When ultrasound is enabled, acoustic sensitizers can effectively produce reactive oxygen species to kill bacteria and accelerate virulence clearance. In vivo optical imaging has shown that an antibody-driven nanotrap can successfully locate MRSA infections and accurately distinguish between lesions and sterile inflammation [50]. This led to the first combination of antimicrobial sonodynamic therapy and antivirulent immunotherapy, providing a powerful new approach to anti-MDR bacterial infections for antibiotic-free nanotherapeutics (Figure 5).

### 3.4. Electromagnetism-Based Nano-Biosensors

Magnetic nanometer-sized particles refer to magnetic particles with particle sizes between 1 and 100 nm, which have the qualities of quantum size effect, surface effect, as well as characteristics of various traditional nanomaterials. Magnetic nanomaterials have outstanding features. They are a kind of nanomaterial with the benefits of superparamagnetization and magnetic conductivity of magnetic substances. In the meantime, they have the wide advantages of simple preparation process, good biocompatibility, and strong surface activity. Relevant technical support for enrichment and separation about bacteria in complex substrates and direct testing of low-abundance bacteria according to clinical specimens could be provided through magnetic nanomaterials [51]. One type of magnetic nanophase material that has been widely studied is magnetic iron oxide (Fe_3_O_4_) nanometer-sized particles. They have surface effects and superparamagnetism. Magnetic Fe_3_O_4_ nanoparticles have a good application prospect in biomedicine, sewage therapy, and other fields. For example, the surface modification of Fe_3_O_4_ can impart specific functions, like adsorption and separation, via an external magnetic field at room temperature. In addition, Fe_3_O_4_ nanoparticles have also been widely used in food and water monitoring. The use of magnetic solids as adsorbents for preconcentration of different analytes from complex matrices, and the thioflavin T aptamers for the development of light-up probes in selection and characterization, has been reported [52,53].

#### 3.4.1. Magneto-Controlled Micromotor

This is a new, magnetically controlled, multifunctional micromotor, the main application scenarios of which are bacterial biofilms as well as bacterial infections. H_2_O_2_ is the fuel and MnO_2_ is the catalyst. Because of the presence of H_2_O_2_, the magnetically controlled multifunctional micromotor can propel itself through the produced oxygen microbubbles, thus drilling into the extracellular polymeric substances (EPSs) of the biofilm then completely destroying it under the assistance of bubbles. Ultimately, unprotected bacteria are not spared and will be killed by the highly toxic •OH produced. This can effectively eliminate microbial infection in microchannels in a short time (within 10 min) and has application prospects in clinical medicine (especially in large-scale complex infection sites) [54]. So, this precisely controlled and deeply permeable micro/nano hybrid multi-functional motor has enhanced antibacterial activity and powerful function against refractory biofilm infections.

#### 3.4.2. Magnetic Cantilevers

The magnetic cantilever does not stand alone, as it needs to be grafted onto a substrate, which we can control remotely. Based on the principle of electromagnetism, when exposed to an alternating magnetic field, a magnetic cantilever deflects vertically from its initial site, autonomously as well as periodically. However, it should be noted that its frequency is not too high (0.16 Hz), and it is generally very low. Surprisingly, because of the above deflection and beating of the magnetic cantilever beam, it indeed effectively prevented the adhesion of bacterial biofilms and prevented an increase in bacterial biofilms in later periods. The researchers’ experimental data on liquid cultures of *E. coli* showed a significant reduction in the formation of bacterial biofilms, up to 70% [55]. This is because the magnetomechanical drive of microstructure construction is indeed efficient in keeping its from, forming bacterial biofilms.

#### 3.4.3. Magnetotactic Bacteria

Magnetotactic bacteria are a variety of strange magnetosensitive bacteria. They could move in the direction of a magnetic area. The magnetic substance magnetite magnetosomes in magnetotactic bacteria could be seen through electron microscopy [56]. Chen constructed an *S. aureus* isolation system by modifying a rabbit anti-MO-1 polyclonal antibody on the surface of magnetotactic bacteria MO-1 cells. This study is the first to demonstrate bacterial microrobots carrying pathogens; they could carry *S. aureus* to an assigned point in charge of the magnetic area and, more importantly, it reflects the great potential of using magnetotactic bacteria to develop magnetic-guided, autopropelled microrobots for pathogen isolation, which laid the foundation for the next detection of pathogenic bacteria. In subsequent studies, the killing effect of *S. aureus* was investigated using MO-1, and a remarkable bactericidal effect was achieved in animal experiments through alternating magnetic-field hyperthermia and the mechanical force of an oscillating magnetic field [57,58,59].

#### 3.4.4. Magnetic Liquid Metal Nanoparticles

**Figure 6:**
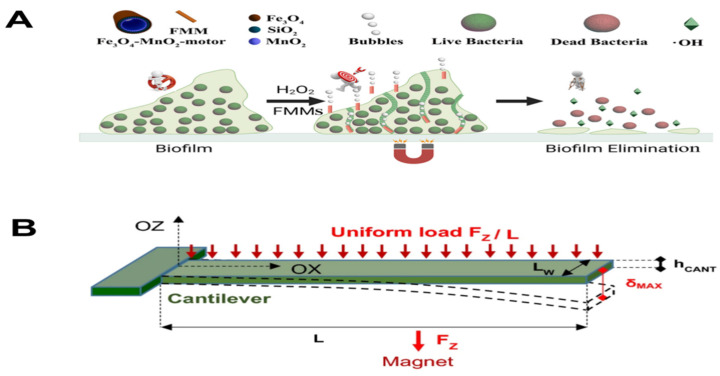
Schematic illustration of multifunctional micromotors for biofilm elimination [54] (**A**). Sketch of the cantilever bending, subjected to the uniformly distributed magnetic force [55] (**B**).

A team investigating the antibacterial potential of magnetic liquid metal nanoparticles provided a proof-of-concept investigation into the use of magneto-responsive gallium-based liquid metal (LM) droplets as antibacterial materials, which can physically damage, disintegrate, and kill pathogens within a mature biofilm. When exposed to a low-intensity magnetic field, these nanometer-sized droplets change shape and form sharp edges. When droplets come into contact with a bacterial biofilm, their movement and nanosharp edges disrupt the biofilm and physically destroy the bacterial cells. In a new study, the team tested the effectiveness of the technique against two types of bacterial biofilms (Gram-positive and Gram-negative). After 90 min of exposure to the liquid metal nanoparticles, both biofilms were destroyed, and 99% of the bacteria died. Importantly, lab tests have shown that these bacteria-killing droplets do not affect human cells [60,61] (Figure 6).

### 3.5. Photothermal Nano-Biosensors

Photothermal therapy (PTT) receives broad widespread attention and research as a non-intrusive and selective technique for diagnosing and treating bacteria. It is a smart technology, mainly based on physical means and supplemented by chemical methods [62,63]. A microorganism is a living body with a cellular structure, and heating will denature its protein until death. The sterilization technology using this principle is known as heating sterilization technology.

#### 3.5.1. Photothermal Ablation

Wu demonstrated a rapid and broad-spectrum antibacterial strategy through photothermal ablation using MXene and light. This is a fast, all-round, and multi-level spectral antimicrobial photothermal nanotechnology. Under 808 nm light, Ti_3_C_2_ MXenes had a very significant antibacterial effect within 20 min, proving effective against 15 kinds of bacteria. This new photothermal method has great potential and, in addition, the rapid antibacterial strategy works for MRSA biofilms by damaging the structures as well as killing bacteria in biofilms [64]. This study broadens the potential applications of photothermography and provides a way to physically destroy bacteria and biofilms without developing drug resistance.

#### 3.5.2. Hybrid Coating

Zhao developed a method of surface functionalization for multifunctional antibacterial uses. The functionalized polyurethane (PU, an extensively used biomedical material for hernia recovering) surface (PU-Au-PEG) with inherent antifouling and photothermal bactericidal properties was readily prepared based on a near-infrared (NIR)-responsive organic/inorganic hybrid coating, which consists of gold nanorods (Au NRs) and polyethylene glycol (PEG). The PU-Au-PEG showed high efficiency to stop the adhesion of bacteria and exhibited effective photothermal bactericidal properties under 808 nm NIR irradiation, especially a fight with MDR bacteria. In addition, the PU-Au-PEG can inhibit biofilm formation in the long term. The biocompatibility of PU-Au-PEG was also demonstrated by cytotoxicity and hemolysis tests [65,66].

#### 3.5.3. Bacterial Affinity Photothermal Carbon Dots

Bacterial affinity photothermal carbon dots (BAPTCDs) that targets MurD ligase catalyze the synthesis of peptidoglycan (PG) by bacteria. The photothermal carbon point has excellent performance and high specificity and sensitivity against bacteria. Because of its special, chiral structure, it has the function of targeting bacteria. By competing with D-glutamate and binding with MurD ligase at the same time, the biological activity of the enzyme is significantly reduced, thus inhibiting the synthesis of the bacterial wall and improving the accuracy of laser treatment for bacteria. Because of the interweaving of the above various effects, the photothermal carbon point’s antibacterial effect for this targeted bacteria is outstanding [67,68].

#### 3.5.4. Intelligent Hybrid Hydrogels

**Figure 7:**
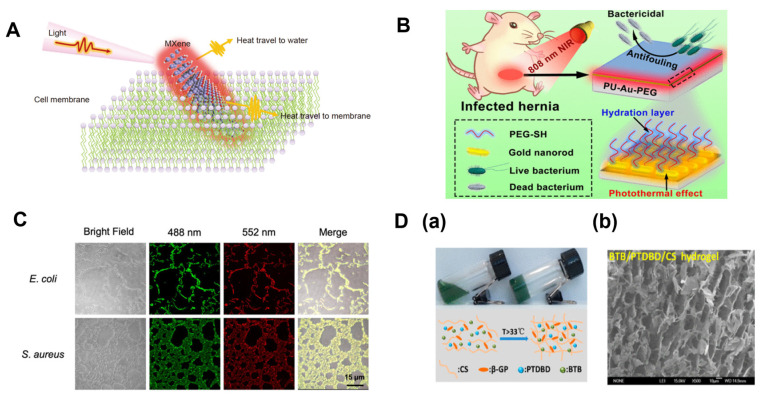
Antibacterial mechanisms of MXene with light [64] (**A**). Schematic Illustration of NIR-Responsive PU-Au-PEG surface with antifouling and photothermal bactericidal properties [65] (**B**). Confocal microscopy images of *E. coli* ATCC 700,926 and *S. aureus* ATCC 29,213 treated with BAPTCDs [67] (**C**). (**a**) Photograph of BTB/PTDBD/CS hydrogel in sol and gel states and schematic illustration of gelation progress; (**b**) representative SEM image of BTB/PTDBD/CS hydrogel [69] (**D**).

Wang synthesized a smart hydrogel that integrates in situ visual diagnosis of bacterial infections with photothermal therapy. Its main antibacterial mechanism is thermotherapy, by simply and subtly incorporating Ph-sensitive Bromothymol blue (BTB) and a conjugated polymer (called PTDBD) that absorbs near-infrared (NIR) into a thermosensitive hydrogel based on chitosan (CS). The synthetic BTB/PTDBD/CS hydrogel can diagnose the acidic microenvironment of *S. aureus* by displaying visual color changes to form biofilms and infected wounds. After rapid diagnosis, the hydrogel can immediately treat the infected site by local high temperature under NIR laser (808 nm) irradiation, even under stubborn biofilms that are difficult to eradicate [69] (Figure 7).

### 3.6. Mechanobiology-Based Nano-Biosensors

Over the last several years, an increasing amount of research has proved that the extracellular matrix (ECM) physical and mechanical microenvironment (such as fluid shear force, osmotic pressure, mechanical strain, interfacial sticking with extracellular matrix stiffness) acts a key model for managing both the normal biology and diseased state features and behaviors of bacteria and host cells [70,71].

#### 3.6.1. Extracellular Matrix Stiffness Regulation

Bacteria are more willing to stick to and attack epithelial cells, especially in places with higher traction strength as well as relative location. ECM stiffness is highly controllable, and under its control, the spatial distribution of bacteria during invasion can be changed, mainly due to the regulation of the F-actin cytoskeleton arrangement in host cells. By targeting ECM stiffness, its main regulation, namely cytoskeleton coordination, alters the course of bacterial infection as well as bacterial resistance [72]. This study not only revealed the key role of matrix stiffness in the regulation of bacterial infection but also provided a new way to improve the clinical antibiotic therapy of multidrug-resistant bacteria from the perspective of mechanobiology.

#### 3.6.2. Three-Dimensional Extracellular Matrix Rigidities

**Figure 8:**
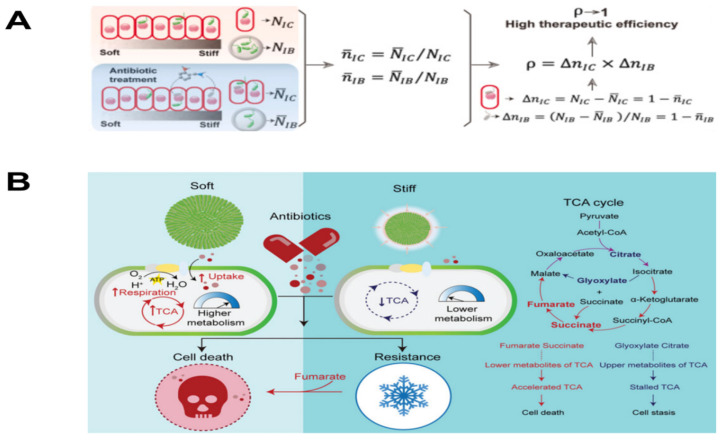
Intracellular accumulation of antibiotics and therapeutic efficacy regulated by matrix stiffness [72]. (**A**) ECM rigidities modulated the TCA cycle and (**B**) antibiotic resistance of 3D confined bacterial microcolonies [73].

Han discovered a high-throughput antibiotic sensitivity testing (AST) platform, where it was proved that 3D ECM rigidities significantly adjust their resistance to diverse antibiotics. The microcolonies in 3D ECM with human tissue-specific rigidities varying from 0.5 to 20 kPa show a ≈ 2–10,000-fold increase in minimum inhibitory concentration, depending on the types of antibiotics. Researchers subsequently identified that the increase in 3D ECM rigidities results in the downregulation of the tricarboxylic acid (TCA) cycle, which is in charge of enhanced antibiotic resistance [73] (Figure 8).

### 3.7. In Situ Bio-Assembly Nano-Biosensors for Bacteria-Related Disease Theranostics

**Figure 9:**
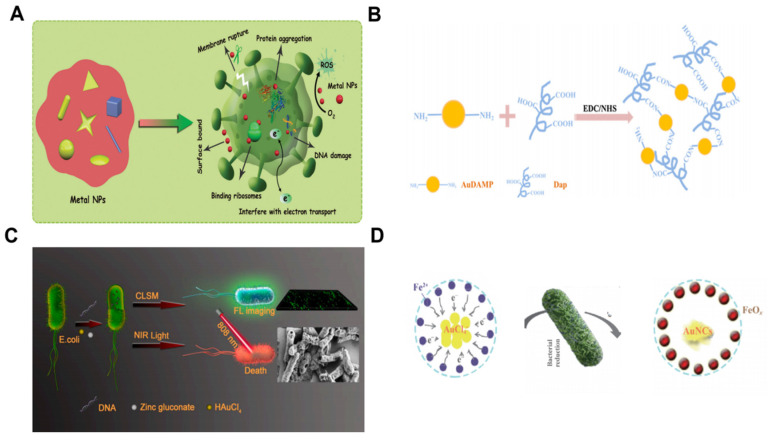
Schematic diagram of the antibacterial mechanism of metal nanoparticles [27] (**A**). Schematic illustrations of the conjugation strategy for antibacterial Au NCs and Dap, conjugation-induced AIE enhancement, and antibacterial synergistic effect [76] (**B**). Schematic illustration of the in situ bioresponsive self-assembled metal NCs complexes in *E. coli* cells. CLSM: confocal laser scanning microscope [77] (**C**). Characteristics of in situ bio-self-assembled NCs [78] (**D**).

In situ synthesis technology is a new method for the accurate target labeling and imaging of malignant tumor cells/tissues/bacteria by using chemical means (adding metal elements) to biosynthesize functional nanocluster probes in vivo [74]. These include fluorescence in situ hybridization (FISH) technology. It is a relatively new technology for the quantification of microbial communities in biofilms and also for the determination of the spatial distribution of microbial populations. It does not require the extraction of nucleic acids, as previous techniques do, and fluorescent oligonucleotide probe labeling could be applied for in situ hybridization. The probes can be labeled with different fluorescent dyes, and each fluorescent agent has different excitation and emission spectra. Therefore, two or more probes can be used simultaneously to detect different populations [75]. In situ synthesis can not only increase the permeability of antimicrobials on the biofilm but also destroy the integrity of the biofilm through physical or biochemical processes such as photothermal conversion. For example, Zheng et al. proposed a highly effective antibacterial hybrid obtained by covalently coupling gold nanoclusters with the antimicrobial peptide datomycin. This synthesized conjugated structure not only has a significantly enhanced synergistic effect but also inherits the inherent properties of the above two preparations. This conjugated structure can produce pores in the membrane through the action of local daptomycin, which could effectively destroy and wipe out the bacterial biofilm [76,77]. Guo et al. used in situ synthesis technology to build bioresponsive nanocomposite materials targeting bacterial bioimaging and disinfection and a multifunctional nanoheating platform based on the unique microenvironment of biofilms. When bacteria are observed, the microenvironmentally responsive nanoclusters can effectively sterilize bacteria due to electrostatic effects, cell membrane destruction, inhibition of biofilm formation, and ROS accumulation [78]. Specific interactions like these with bacteria make in situ synthesis an interesting tool for diagnosing and treating bacteria with precision [79] (Figure 9).

## 4. Conclusions

Bacterial infections, along with drug resistance and the formation of bacterial biofilms, can cause acute, chronic, refractory, and even incurable diseases. Today, many antimicrobials are commonly used to fight bacterial infections, but irrational antimicrobial misuse can also lead to inefficient biofilm control and the spread of resistance.

Traditional biological detection and antibiotic drug therapy methods require a lot of money, time, and effort. The latest multiple nanotechnology approaches can be readily utilized in targeting MDR bacteria and eliminating antimicrobial biofilms. The benefits are numerous, such as high sensitivity and strong specificity, full-time monitoring, time-saving and labor-saving characteristics, etc. To our surprise, some of these applications are already being used in clinical trials.

In the next few years, the development of new biofilm-specific antibiotics will be of great help in controlling biofilm infections. It is important to take biofilm phenotypes into account when designing and testing new drug candidates. As mentioned above, several approaches are still in the early stages of development. For example, the use of engineered antimicrobial peptides as antibacterial agents offers a new method to treat bacterial resistance and biofilm infections. What’s more, liposomes, polymer particles, and dendrimers from novel drug delivery systems could improve the permeability of antibacterial drugs in bacterial biofilms, thereby enhancing their efficacy, capacity, and potential.

Fortunately, the system of applying multiple nanotechnology approaches to accurately diagnose and treat bacteria is constantly improving and maturing, and a growing number of productive nanosystems could be applied to medical diagnosis and the therapy of bacteria in the near future. Meanwhile, in order to ensure good bactericidal effects from the nanosystem, it is also necessary to evaluate their cytotoxicity and reduce the toxic effects by changing the size, shape, surface charges, and so on. At the same time, for engineered nanosystems with human cells, tissues, and organs, quantification and standardized production should be further explored. As for the next step, how to improve and promote the industrial level of the above bacteriological treatment efficiency is still a significant challenge that needs to be urgently addressed in the near future.
